# Detection of Carbapenem-Resistant Genes Among Clinical Isolates of Acinetobacter baumannii in a Tertiary Care Hospital

**DOI:** 10.7759/cureus.85498

**Published:** 2025-06-07

**Authors:** Gaurav Verma, Subham Ravi Nayak, Shradha Smriti, Liza Das, Subhra Snigdha Panda, Dipti Pattnaik, Nipa Singh, Ashok K Praharaj, Sukanta Tripathy

**Affiliations:** 1 Microbiology, Kalinga Institute of Medical Sciences, Bhubaneswar, IND; 2 Transfusion Medicine, Kalinga Institute of Medical Sciences, Bhubaneswar, IND

**Keywords:** acinetobacter baumannii, carbapenem-resistance, gene, new delhi metallo-β-lactamase, real-time pcr

## Abstract

Background

*Acinetobacter baumannii*, an opportunistic pathogen associated with healthcare-associated infections, poses a major clinical challenge due to its ability to develop resistance to multiple drugs, particularly carbapenems.

Objective

The aim of the study was to detect the presence of different carbapenem resistance genes in clinical isolates of *Acinetobacter baumannii*.

Materials and methods

A cross-sectional study was conducted from March 2023 to December 2023 in the Department of Microbiology of a tertiary care hospital, India. A total of 107 non-duplicate clinical isolates of *Acinetobacter baumannii* resistant to carbapenems were screened and collected. Bacterial species identification and antimicrobial susceptibility testing were performed using the VITEK 2 automated system (AST N405 panel). Carbapenem resistance genes were detected by real-time polymerase chain reaction (q-PCR) targeting blaNDM, blaOXA48, blaVIM, blaKPC and blaIMP genes using multiplex TRUPCR UTI AST Panel Kit. Data analysis was performed using Microsoft Excel (Microsoft Corp., Redmond, WA, USA), and the frequency of detected genes was expressed in percentages.

Result

Out of the 107 carbapenem-resistant *A. baumannii* isolates, 20 isolates were subjected to molecular detection of carbapenem-resistant genes. Out of the 20 isolates tested for resistance genes, *blaNDM *was detected in 35% (7/20) of the isolates, while *blaOXA-48 *was found in 30% (6/20). blaNDM+blaOXA-48 were found to co-exist in 20% (4/20) of the isolates. blaIMP+blaKPC, blaOXA48+IMP, blaIMP+blaNDM genes were found in one isolate each (5%, 1/20).

Conclusion

The detection of *blaNDM* and *blaOXA-48* genes in carbapenem-resistant *Acinetobacter baumannii* highlights the growing threat of multidrug-resistant pathogens in clinical settings. The co-existence of these resistance genes underlines the need for routine molecular surveillance and stringent antimicrobial stewardship to prevent the spread of such high-risk clones.

## Introduction

*Acinetobacter baumannii* is a non-fermenting, Gram-negative opportunistic pathogen that has emerged as a significant cause of healthcare-associated infections (HAIs) globally. It primarily affects critically ill and immunocompromised individuals, especially those in intensive care units (ICUs), and is responsible for a broad spectrum of infections including ventilator-associated pneumonia, bloodstream infections, urinary tract infections, wound infections, and meningitis. These infections are associated with prolonged hospital stays, increased healthcare costs, and high morbidity and mortality rates. The World Health Organization (WHO) classified carbapenem-resistant *A. baumannii* (CRAb) as a “critical priority pathogen” in 2017, which emphasizes the urgent need for the development of new antimicrobial agents and improved diagnostic methods [1].

A major concern with *A. baumannii* is its extraordinary ability to develop resistance to a wide range of antibiotics through both intrinsic and acquired mechanisms. Among the most alarming trends is resistance to carbapenems, which are often used as last-resort antibiotics for treating multidrug-resistant Gram-negative bacterial infections [2]. Carbapenem resistance in *A. baumannii* is primarily mediated by the production of carbapenem-hydrolyzing β-lactamases, known as carbapenemases. These enzymes belong to Ambler class D (oxacillinases) and class B (metallo-β-lactamases). The production of carbapenemases has a significant effect on the utilization of carbapenems, leading to elevated rates of treatment failure [3].

In India, the prevalence of carbapenem-resistant *A. baumannii* has been reported to range from 40% to 75%, with regional variations and increasing trends in tertiary care settings [4]. Carbapenem resistance in *A. baumannii* is primarily caused by Ambler Class D and Class B carbapenemases [5]. In lactose, non-fermenting bacteria (such as *A. baumannii* and *Pseudomonas aeruginosa*) have various mechanisms that contribute to carbapenem resistance like porins and efflux pumps. The predominant mechanism of CRAb is the production of class D carbapenemases, such as blaOXA-23-like, blaOXA-24-like, blaOXA-58-like, and blaOXA-48-like. *A. baumannii* strains producing blaOXA-23-like carbapenemases, which are responsible for outbreaks, have been documented in various regions across the world [6-8].

Despite the clinical significance, molecular surveillance data from many parts of India, especially the eastern region, remain sparse. Therefore, the objective of the present study was to investigate the molecular profile of carbapenem-resistant *A. baumannii* clinical isolates, with a specific focus on identifying key carbapenem resistance genes such as blaNDM, blaOXA-48, blaVIM, blaKPC and blaIMP.

## Materials and methods

This cross-sectional study was conducted from March 2023 to December 2023 in the Department of Microbiology, Kalinga Institute of Medical Sciences, Bhubaneswar, Odisha, India. Ethical approval for the study has been obtained from the Institutional Ethics Committee (reference no: KIIT/KIMS/IEC/1344/2023).

Sample collection and procedure

The various clinical samples were obtained from different departments. The samples (such as ET, Blood, Tissue, Pus, Wound swab and others (BAL, Swabs, CVP)) were sent to the central laboratory of the Department of Microbiology, KIMS, KIIT DU. The samples were processed as per standard protocol. The samples that showed positive bacterial culture were further processed for identification and antibiotic susceptibility testing (AST). It was done by the automated VITEK 2 compact system (bioMerieux, Marcy-l'Etoile, France). The isolates, which were identified as *A. baumannii* and were resistant to at least one carbapenem antibiotic (ertapenem, imipenem, meropenem) as per the Clinical Laboratory Standard Institute (CLSI) 2023 guidelines, were included in the study.

A total of 622 *A. baumannii* isolates were obtained during the study period, out of which CRAB constituted 107 (17.2%, 107/622). Real-time polymerase chain reaction analysis was done only for 20 representative CRAB isolates.

Antibiotic susceptibility testing

The test organism was inoculated onto the agar at a concentration of 0.5 McFarland standards (HiMedia McFarland standard set, R092-1No; HiMedia, Mumbai, India) ensuring semi-confluent growth following the recommendations of the Clinical and Laboratory Standards Institute (CLSI 2023). To confirm the results, the VITEK-2 system (bioMérieux, Marcy-l’Etoile, France) was utilized, employing the 405 AST panel. The CLSI guidelines were followed for the ASTs against piperacillin-tazobactam (100/10 µg), cefotaxime/ceftriaxone (30/30 µg), ceftazidime (30 µg), cefepime (30 µg), doripenem (10 µg), imipenem (10 µg), meropenem (10 µg), tetracycline (30 µg), minocycline (30 µg), polymyxin B, colistin, gentamicin (10 µg), tobramycin (10 µg), amikacin (30 µg), ciprofloxacin/levofloxacin (5 µg) and trimethoprim/sulfamethoxazole (1.25/23.75 µg). The quality controls used were American Type Culture Collection (ATCC) strains E. coli 25922.

Simplified carbapenem inactivation method (sCIM)

The simplified carbapenem inactivation method (sCIM) is an improved version of the modified carbapenem inactivation method (mCIM) that incorporates enhanced experimental procedures. Unlike the mCIM, where the antibiotic disk is incubated in the organism culture media for 4 hours, the sCIM involves directly smearing the organism to be tested onto an antibiotic disk. To conduct the sCIM for Enterobacteriaceae, we inoculated a Mueller-Hinton agar (MHA) plate with a 0.5 McFarland suspension of E. coli ATCC 25922, prepared via the direct colony suspension technique. For *A. baumannii* and *P. aeruginosa*, we used a diluted 1:10 suspension of the same E. coli standard in saline. The inoculation on the MHA plate also followed the direct colony suspension method. After inoculation, the plates were allowed to dry for 3 to 10 minutes. Next, we transferred 1 to 3 overnight colonies of the test organisms, cultured on blood agar, onto an imipenem disk (10 µg; HiMedia, Mumbai, India).

Molecular detection of carbapenem resistance genes

Twenty representative isolates were selected for molecular detection. DNA extraction from isolates was performed using a commercially available spin column, following the instructions provided by the manufacturer (TRUPCR nucleic acid extraction kit, TRUPCR 3B BlackBio Biotech India Ltd., Bhopal, India). The purity and concentration of the bacterial DNA were assessed by measuring 1 μl of the eluted DNA using a Nanodrop Spectrophotometer, which measures absorbance at 260 and 280 nm (Multiskan Sky, Thermo Scientific, USA). The eluted DNA was then subjected to amplification for the detection of carbapenem-resistance target genes blaNDM, blaKPC, blaIMP, blaVIM, and blaOXA-48 using probe-based real-time PCR (QuantStudio 5, Applied Biosystems, Waltham, MA, USA). The blaNDM gene was detected in 35% (7/20) of the samples, followed by the blaOXA-48 gene in 30% (6/20). Co-occurrences of genes were observed in blaNDM and blaOXA-48 were found together in 20% (4/20) of the samples, while blaIMP with blaKPC was detected in 5% (1/20), blaOXA-48 with blaIMP in 5% (1/20) and blaIMP with blaNDM in 5% (1/20) of the samples. The real-time PCR conditions included an initial denaturation step at 94°C for 10 minutes, followed by denaturation at 94°C for 15 seconds, annealing at 60°C for 45 seconds, and extension at 72°C for 15 seconds, repeated for 38 PCR cycles.

Result Analysis

The results were interpreted based on the Ct value of the amplified product, using the cutoff value specified in the TRUPCR carbapenem-resistant detection kit (LOT EXT TNA/2023/05, REF 3B347), and analyzed using the Design and Analysis Software for the QuantStudio 5 Real-Time PCR System.

## Results

Out of 622 *A. baumannii* isolates, carbapenem resistance was detected in 107 (17.2%) isolates. One hundred seven non-duplicate *A. baumannii* isolates from various clinical samples were included in the study. The prevalence of carbapenem resistance was higher in ICUs (74.4%, 80/107) followed by wards (25.2%, 27/107). In males, the prevalence of CRAB cases was higher (63.6%) than females (36.4%). The mean age of patients was 51.1 years with a median of 54 years and standard deviation of 19.7.

**Figure 1:**
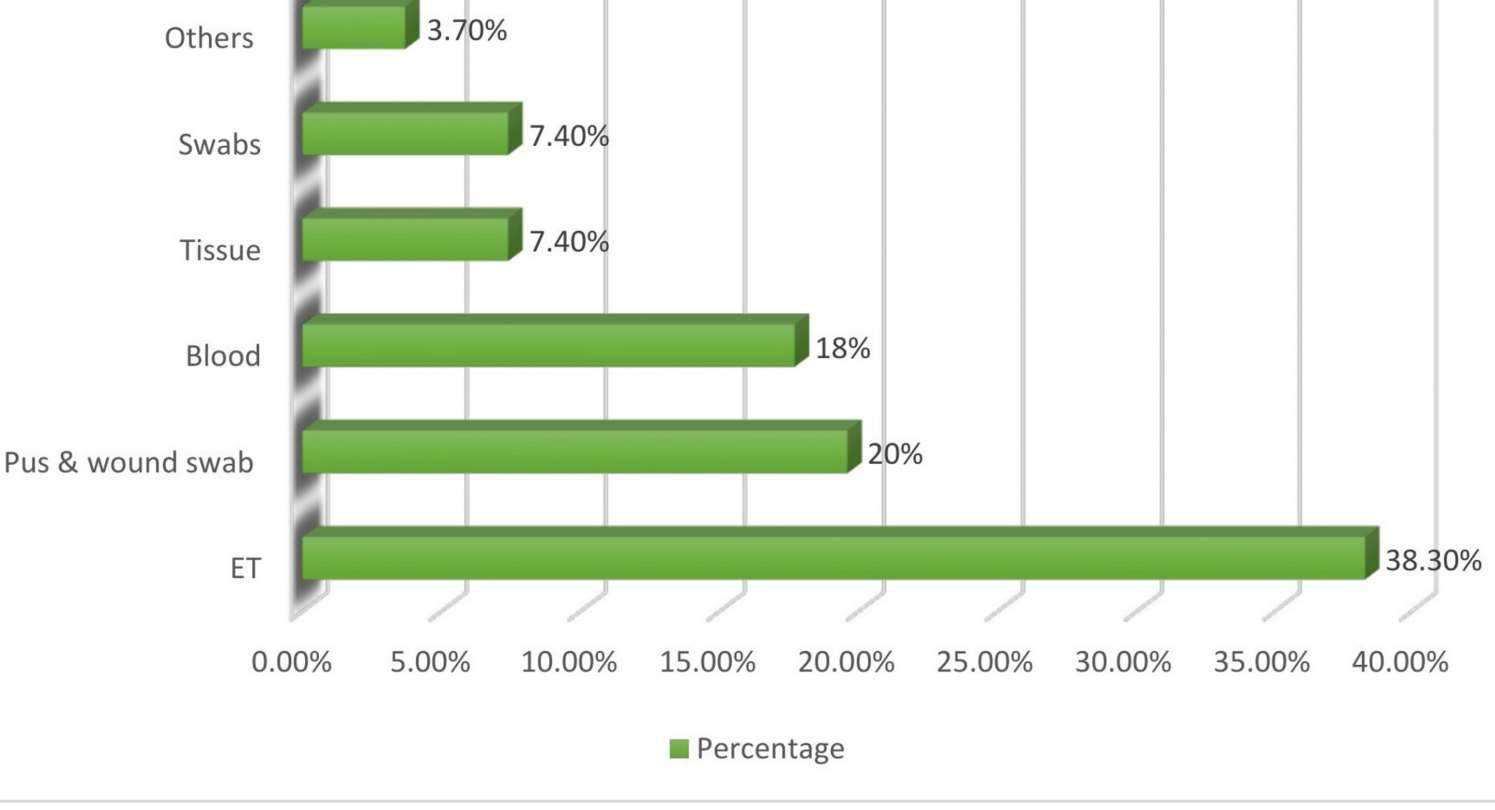
Sample wise distribution of carbapenem-resistant A. baumannii ET: endotracheal tube; swabs: vaginal swab, rectal swab.

The maximum number of isolates was recovered from respiratory secretions (38.3%, 41/107) followed by pus and wound swabs (19.6%, 21/107), blood (17.7%, 19/107), swabs (7.4%, 8/107), tissue (7.4%, 8/107) and others (3.7%, 4/107) listed in Figure 1.

**Figure 2:**
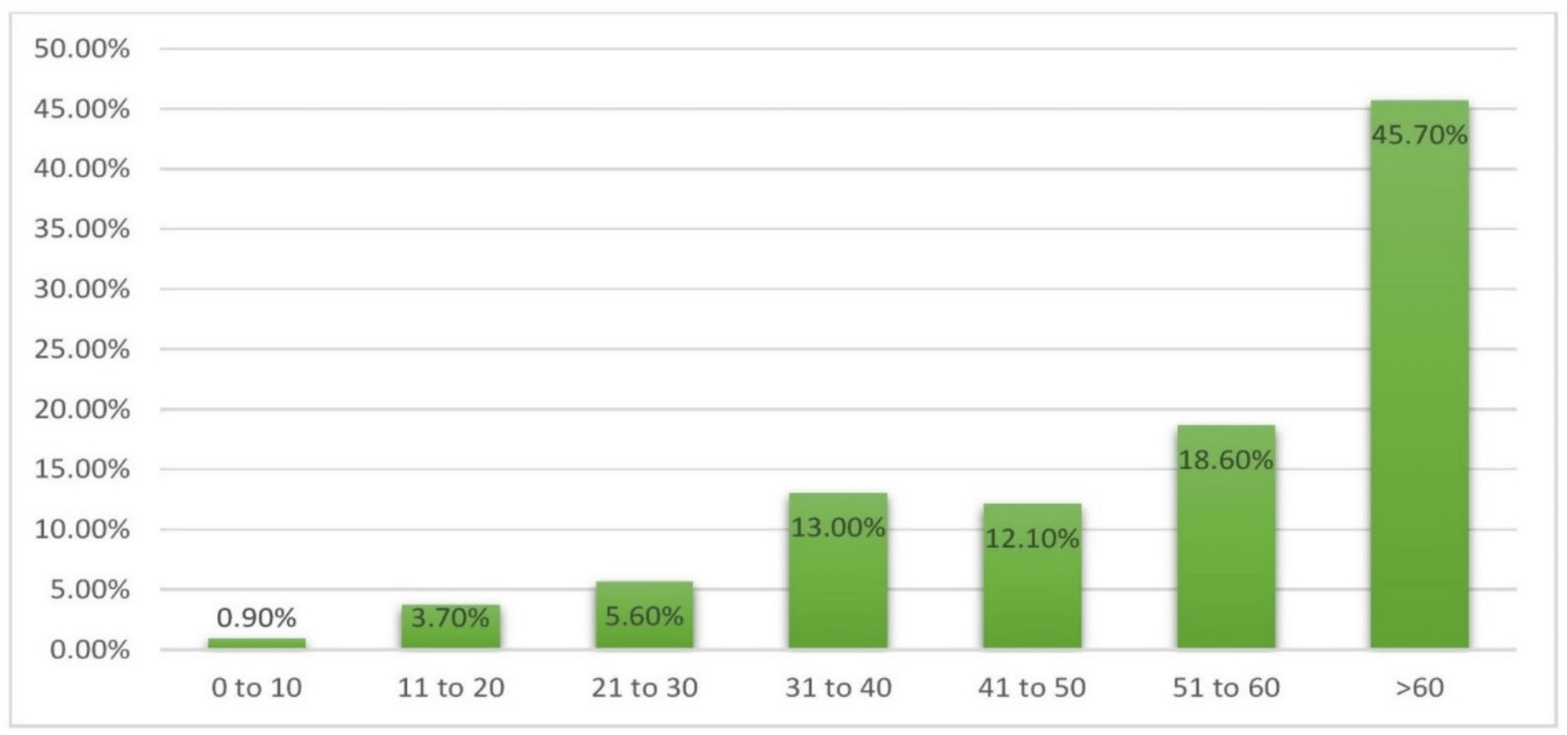
Age wise distribution of carbapenem-resistant A. baumannii

Among the 107 carbapenem-resistant *Acinetobacter baumannii* isolates, the highest number of CRAB cases were observed in the patients aged over 60 years (45.7%, 49/107), followed by 51-60 years (18.6%, 20/107), as shown in Figure 2.

The prevalence of carbapenemase production among carbapenem-resistant *A. baumannii* by the sCIM test was 83.1%, whereas 13% (14/107) were shown negative to sCIM and 3.7% (3/107) were carbapenemase indeterminate. The distribution of carbapenemase production is shown in Tables 1, 2. Table 3 illustrates the antibiotic susceptibility pattern of 107 carbapenem-resistant *A. baumannii*.

**Table 1:** Frequency of sCIM test in ICU and Wards sCIM: Simplified Carbapenem Inactivation Method

Test	ICUs (n=81)	Wards (n=26)		Total (N=107)
sCIM positive	67 (82.7%)	22 (84.6%)		89 (83.1%)
sCIM negative	10 (12.3%)	04 (15.3%)		14 (13.0%)
sCIM indeterminate	04 (4.9%)	-		04 (3.7%)

**Table 2:** Demographic distribution of carbapenemase production *The association between gender and location is considered to be not statistically significant (p-value = 0.34).

Gender	ICUs (n=81)	Wards (n=26)	Chi-Square	P-value
Male (n=68)	49	19		
Female (n=39)	32	07	1.3454	0.34
Total (107)	81	26		
Age (Years)	Carbapenemase Positive (sCIM +ve )	Carbapenemase Negative (sCIM –ve)		Carbapenemase Indeterminate
0-10	01	-		-
11-20	03	-		01
21-30	05	01		-
31-40	12	02		-
41-50	10	04		-
51-60	18	01		-
>60	40	06		03

**Table 3:** Antimicrobial susceptibility profile of carbapenem-resistant A. baumannii isolates (N=107)

Antibiotics	Resistant	Percentage %	Sensitive	Percentage %
Piperacillin/Tazobactam	106	99.0	01	01
Ceftazidime	100	93.4	07	6.5
Cefoperazone/Sulbactam	101	94.3	06	5.6
Cefepime	99	92.5	08	7.4
Imipenem	106	99.0	01	01
Meropenem	105	98.1	02	1.8
Amikacin	102	95.3	05	4.6
Gentamicin	100	93.4	07	6.5
Ciprofloxacin	99	92.5	08	7.4
Levofloxacin	76	71.0	31	29.0
Minocycline	56	52.3	51	47.6
Tigecycline	67	62.6	40	37.3
Colistin	102	95.3	05	4.6
Trimethoprim/Sulfamethoxazole	90	84.1	17	15.8

**Figure 3:**
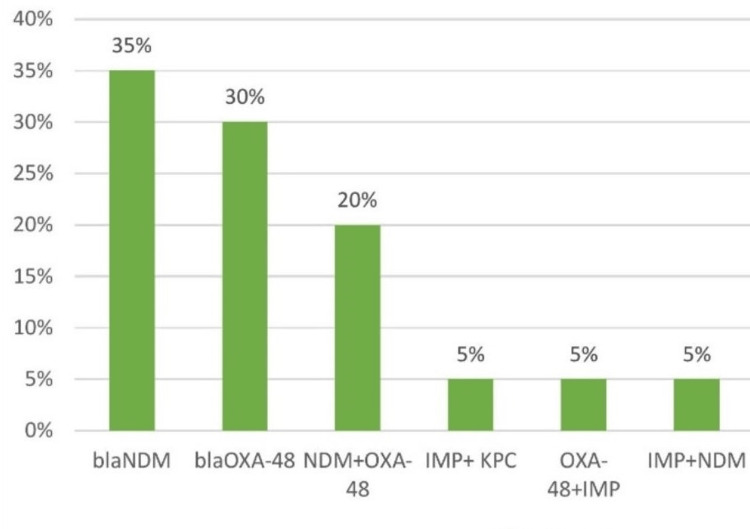
Distribution of carbapenem resistance genes in A. baumannii blaNDM (β-lactamase New Delhi Metallo-β-lactamase); blaOXA-48 (β-lactamase Oxacillinase-48); KPC (Klebsiella pneumoniae carbapenemase); IMP (Imipenemase)

Molecular detection of carbapenem-resistant isolates for the presence of the blaNDM, blaKPC, blaIMP, blaVIM and blaOXA-48 genes in selected isolates was confirmed by Real-time PCR, as shown in Figure 3. Twenty representative isolates were selected for the detection of carbapenem resistance genes by real-time polymerase chain reaction - 35% (7/20) showed the presence of blaNDM gene followed by blaOXA-48 (30%, 6/20), blaNDM + blaOXA-48 gene (20%, 4/20), blaIMP+ KPC gene (5%, 1/20), blaOXA-48+IMP gene (5%, 1/20) and blaIMP+NDM gene (5%, 1/20).

**Figure 4:**
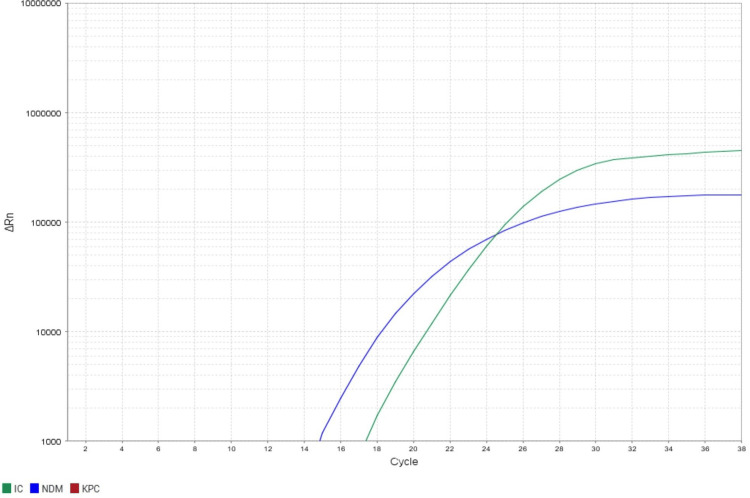
Amplification plot showing detection of blaNDM gene amplification by real-time PCR of representative samples Real-time PCR amplification of representative sample showing amplification of blaNDM gene. Green: internal control, Blue: blaNDM gene target

**Figure 5:**
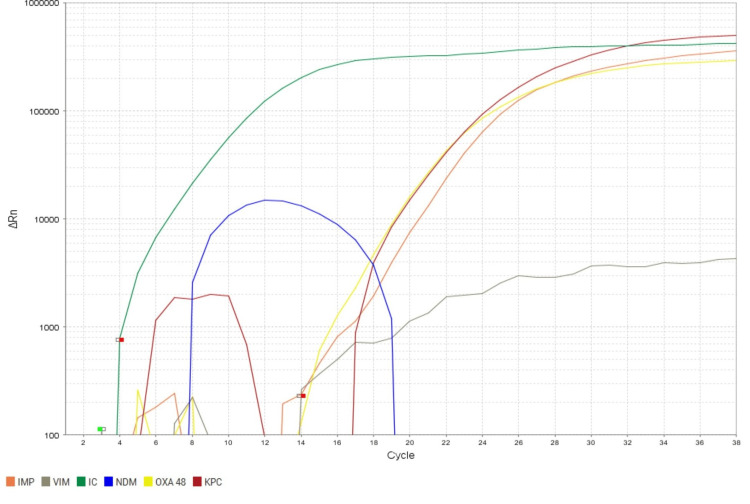
Amplification plot showing detection of multiple gene amplification by real-time PCR of representative samples Green: Internal control; Grey: blaVIM gene; Orange: blaIMP gene; Blue: blaNDM gene; Yellow: blaOXA-48 gene; Red: blaKPC gene

Molecular detection of carbapenem-resistant genes for the presence of the blaNDM, blaKPC, blaIMP, blaVIM and blaOXA-48 gene in all CRAB isolates was confirmed by Real-time PCR, as shown in Figures 4, 5.

## Discussion

In healthcare settings, *Acinetobacter baumannii* acts as an opportunistic pathogen, leading to nosocomial infections. The resistance it develops against carbapenems and other antimicrobial agents adds complexity to the treatment of these infections.

The current study found that 94% of CRAB strains were resistant to third-generation cephalosporins (101/107; 95% CI: 87.7%-97.4%). Specifically, resistance rates were 92.3% for cefepime (99/107; 95% CI: 85.7%-96.3%), 99% for piperacillin/tazobactam (106/107; 95% CI: 94.9%-99.9%), and 84.1% for trimethoprim/sulfamethoxazole (90/107; 95% CI: 76.1%-89.9%). The resistance percentages for the remaining antibiotics varied between 52.3% and 99%, except for colistin, which showed a resistance rate of only 4.6% (5/107; 95% CI: 2.0%-10.3%). Li et al. conducted a study that revealed the presence of blaOXA-51-like in 183 samples (75.00%), blaOXA-23-like in 174 samples (71.30%), blaNDM-1 in 57 samples (23.40%), and blaOXA-58-like in 30 samples (12.30%) [9]. These findings align closely with the research conducted by Anane et al. in 2020 [10,11].

Saranathan et al. conducted another study that found 31% of the strains contained blaIMP-like genes, while 15% had blaNDM-like genes. In addition, Hadjadj et al. analyzed six strains of *A. baumannii*, which included one strain with both blaOXA-23-like and blaOXA-24-like genes, and five strains that had blaOXA-23-like and blaNDM genes [3,10].

The increasing prevalence of carbapenem resistance in *Acinetobacter baumannii* presents a major challenge as carbapenems are the primary treatment for infections caused by this pathogen. Current data indicates that a vast majority of *A. baumannii* strains are resistant to these antibiotics with 99% showing resistance to imipenem and 98% to meropenem. In research conducted by Lowings et al., all multidrug-resistant *A. baumannii* isolates displayed high resistance levels, including 100% to cefotaxime, 89% to ceftazidime, 90% to cefepime, and 86% to both imipenem and meropenem, which stands in contrast to our findings [12].

Our research indicated that *A. baumannii* isolates were primarily identified in respiratory samples, particularly from endotracheal tubes (38.3%), with the highest prevalence of CRAB occurring in ICU settings (75.7%). In comparison, Anane et al., Lowings et al., and Guckan et al. reported isolation rates of *A. baumannii* from ICUs at 41%, 85.2%, and 12%, respectively [11-13].

The presence of carbapenemase-encoding genes, especially OXA-type and blaNDM-1, has been observed in carbapenem-resistant *A. baumannii* across the globe [14]. The first OXA-type enzyme capable of hydrolyzing carbapenems was identified in a clinical strain of *A. baumannii* isolated in 1985 in Edinburgh, UK. In 2004, the blaOXA-23 gene was first reported in Algeria [15]. In our current study, the most common carbapenem-resistant gene was blaNDM (35%, 07/20) followed by blaOXA-48 (30%, 06/20). Seven isolates were found to carry multiple carbapenem-resistant genes, consistent with the study by Santajit et al. [8], which reported the presence of blaOXA-51-like (100%), blaVIM (79.6%), blaIMP (0.58%), blaNDM (12.21%) and blaOXA23-like (93.6%) genes. Similarly, a study by Rose et al. [16] found that 64% of the isolates harbored both the blaOXA-23 and the intrinsic blaOXA-51-like genes.

Additionally, in 1% of the isolates, the blaNDM-1 gene coexisted with the blaOXA-51-like gene, while 27% of the isolates reported the presence of both blaOXA-23 and blaNDM-1 genes. Previous studies in India [17] have also documented the co-occurrence of the blaNDM-1 gene and the blaOXA-23 gene. Such genetic combinations significantly enhance the bacterium’s capacity to resist multiple classes of antibiotics and reduce treatment efficacy.

The presence of blaOXA-48, blaIMP, blaVIM, and blaNDM genes can potentially be observed on either the chromosome or plasmid. This suggests that the occurrence of other resistance genes may be a result of different genes coexisting within the same strain. Another possibility is the existence of a polyclonal population of *A. baumannii* originating from the same sample. These populations cannot be visually identified without the aid of magnification. Despite the limited number of strains analyzed, our research provides evidence that the simultaneous presence of carbapenemases in *A. baumannii* might be associated with multiple clones [18-20].

The results obtained from this study have not only provided valuable insights into the susceptibility of antimicrobials but have also shed light on the prevalent resistance mechanism among CRAB. This information plays a vital role in preventing the emergence of multidrug-resistant (MDR) *A. baumannii* in healthcare facilities and assists healthcare providers in making well-informed decisions regarding the initial treatment of patients [21]. To effectively address the increasing prevalence of carbapenem-resistant *A. baumannii*, it is essential to adopt regular and affordable screening techniques for identifying carbapenemase production in healthcare settings. This approach will enhance infection control measures and promote responsible antimicrobial usage. Additionally, the interplay of different factors driving the rapid rise of antibiotic resistance in *A. baumannii* in eastern India warrants further research.

This study establishes a correlation between phenotypic and genotypic traits of drug-resistant bacteria. Nonetheless, there are several limitations to consider. Firstly, the bacterial strains were sourced from a single hospital, potentially restricting the genetic diversity reflected in the research. Secondly, sequencing the PCR-amplified antibiotic resistance genes could yield more detailed insights into genetic variations, including single-nucleotide polymorphisms (SNPs), and reveal more intricate relationships. Lastly, expanding the number of bacterial isolates would enhance the statistical robustness and overall credibility of the results.

Our study has some limitations. Although the study analyzed 107 carbapenem-resistant isolates, expanding the sample size could enhance the robustness of the statistical analyses and the reliability of the results, potentially revealing additional resistance patterns and mechanisms. While real-time PCR was used to detect specific carbapenem resistance genes, the lack of whole-genome sequencing limits the ability to explore genetic diversity and co-existing resistance mechanisms, which could highlight subtle genetic variations and provide a more comprehensive understanding of resistance development.

## Conclusions

The findings from the study reveal that carbapenem resistance genes are prevalent among clinical isolates of *A. baumannii*. The resistance is mediated by diverse genetic mechanisms, including the co-expression of beta-lactamase genes. The presence of resistance genes in *A. baumannii* highlights the importance of continuous monitoring to detect their occurrence. The current study emphasizes the importance of screening for drug-resistant genes in carbapenem-resistant *A. baumannii*, as it reveals the presence of multiple carbapenem-resistant genes in clinical isolates.
